# *Plasmodium relictum* MSP-1 capture antigen-based ELISA for detection of avian malaria antibodies in African penguins (*Spheniscus demersus*)

**DOI:** 10.1016/j.ijppaw.2022.08.009

**Published:** 2022-08-29

**Authors:** Xuejin Zhang, Siobhan N.A. Meadows, Tori Martin, Alex Doran, Rachel Angles, Samantha Sander, Ellen Bronson, William H. Witola

**Affiliations:** a Department of Pathobiology, College of Veterinary Medicine, University of Illinois Urbana-Champaign, 2001 S. Lincoln Avenue, Urbana, IL, 61802, USA; b Department of Veterinary Clinical Medicine, College of Veterinary Medicine, University of Illinois Urbana-Champaign, 2001 S. Lincoln Avenue, Urbana, IL, 61802, USA; c The Maryland Zoo In Baltimore, Baltimore, MD, 21217, USA

**Keywords:** *Plasmodium relictum*, ELISA, Anti-malaria antibodies, Captive African penguins

## Abstract

Avian malaria, caused by *Plasmodium* spp. and transmitted by mosquitos, is a leading cause of mortality of captive penguins. Antimalarial drugs are currently used to control infections in penguins. However, the effectiveness of treatment reduces significantly by the time the clinical signs appear, while early and unnecessary treatment interferes with development of protective immunity. Therefore, for suppressing parasitemia without affecting the development of immunity in captive penguins, antimalaria drugs need to be administered at the right time, which requires reliable diagnostic tools that can determine the levels of circulating antimalaria antibodies. In the present study, we have developed an enzyme-linked immunosorbent assay (ELISA) diagnostic assay based on the merozoite surface protein 1 (MSP-1) of *P. relictum* isolate SGS1 to specifically detect and relatively quantify antimalaria antibodies in penguins. We expressed and purified a truncated *P. relictum* isolate SGS1 MSP-1 and optimized its biotinylation and subsequent conjugation to streptavidin alkaline phosphatase for signal generation in ELISA. We tested the assay by analyzing sera obtained from penguins at the Baltimore Zoo, from Spring through Fall, and found that levels of detectable antibodies against MSP-1 varied seasonally for individual penguins, consistent with the expected seasonal variations in avian malaria prevalence. Corroboratively, we analyzed the sensitivity of the assay by titrating positive sera and found that the signal intensity generated was serum concentration-dependent, thus validating the ability of the assay to detect and relatively quantify the levels of antimalaria antibodies in penguin sera.

## Highlights

• ELISA based on MSP1 for detection and quantification of antibodies against *Plasmodium relictum* in birds was developed.
• Assay was validated to detect and quantify levels of antimalaria antibodies in infected penguins' sera.
• Assay detected varied antibody levels against MSP-1 in penguin sera consistent with seasonal variations in malaria prevalence.

## Graphical abstract

**Figure:**
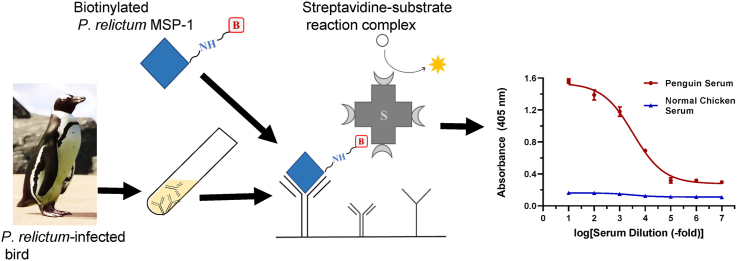

## 1 Introduction

Malaria is a mosquito-transmitted disease caused by protozoan parasites of the genus *Plasmodium*, that parasitize a wide range of vertebrates, from reptiles to mammals (Sato, 2021). Over 200 *Plasmodium* spp. are known to infect birds, but the majority of the infections are asymptomatic (Vanstreels et al., 2015; Grilo et al., 2016). However, penguins (*Spheniscidae*), especially captive penguins in zoos and rehabilitation centers, are highly susceptible to the infection and may develop severe and sometimes fatal disease, (Graczyk et al., 1995; Grim et al., 2003; Bueno et al., 2010; Silveira et al., 2013; Vanstreels et al., 2014; Grilo et al., 2016; Iurescia et al., 2021). It has been reported that the primary cause of mortality for the captive African penguin (*Spheniscus demersus*) population in the Baltimore Zoo (Baltimore, Maryland, USA) is avian malaria (Graczyk et al., 1994a, 1994b).

Since the first report of avian malaria in a king penguin (*Aptenodytes patagonicus*) about nine decades ago, seven *Plasmodium* spp. causing malaria in penguins have been documented, among which *P. relictum* and *P. elongatum* are the most common and pathogenic, causing outbreaks with higher mortality than the other five species (Beier and Stoskopf, 1980). For captive penguins in the northern hemisphere, malaria outbreaks often occur during Summer or early Fall, resulting in as high as 80% cumulative mortality, primarily concentrated in June to October (Vanstreels et al., 2014). Although treatment with antimalarial drugs primaquine and chloroquine can reduce the mortality to as low as 10%, the effectiveness of the treatment is closely associated with the time of the treatment, and decreases dramatically once the first clinical signs appear (Grilo et al., 2016).

Microscopic examination of Giemsa-stained thin blood smears is considered the gold standard test for malaria diagnosis in penguins (Atkinson et al., 2001). However, parasitemia has been shown to be an inadequate diagnosis tool for improving the survival rate of penguins during malaria outbreaks (Grilo et al., 2016). Fatal cases of acute malarial infections in captive penguins with no sign of parasitemia are common in zoos and rehabilitation centers (Graczyk et al., 1995; Atkinson et al., 2001). The sensitivity of the method is low, to the extent that it can miss about 70% of chronic infections as parasites may sequester in tissues and be undetectable. While PCR has significantly higher sensitivity for detecting *Plasmodium* infection than the light microscopic method, it may still fail to detect the infection when insufficient parasite DNA is present in the blood circulation (Grilo et al., 2016). PCR-based methods targeting either parasite mitochondria cytochrome-b (Cytb) gene or conserved ribosomal DNA regions have relatively higher sensitivity than the traditional microscopic-based method, and can distinguish *Plasmodium* spp. (Bell et al., 2015; Schoener et al., 2017). For this reason, PCR and microscopic methods are used together to improve specificity and accuracy of parasite identification, but this turns out to be costly and time-consuming (Krams et al., 2012; Grilo et al., 2016).

An enzyme-linked immunosorbent assay (ELISA) based on the cross-reactivity of antibodies against *P. falciparum* antigens with *P. relictum* and anti-*P. elongatum* antigens was developed for detecting *Plasmodium* infections and monitoring the level of anti-*Plasmodium* antibodies in penguins (Graczyk et al., 1993, 1994c). The *P. falciparum* antigens used included the circumsporozoite protein, the gametocyte antigens and crude red blood cell extracts (Graczyk et al., 1994a). These antigens are not specific to the avian malaria species (*P. relictum* and *P. elongatum*). Further, circumsporozoite antigen used is expressed by the *Plasmodium* sporozoite stage that are transmitted from the mosquito to the host and exist in the host only during the initial early infection stage before transforming into the merozoite stage that are more abundant and sustain the parasitemia throughout the infection. In addition, the gametocytes antigens included in the assay are not abundant. The use of those *P. falciparum* antigens was not based on their specificity for the detection of pathogenic penguin malaria parasites species antibodies, but on their cross-reactivity with antibodies against various *Plasmodium* spp. antigens (Graczyk et al., 1993, 1994c). As such, results that were observed in a prevalence study of malaria infection in penguins on the Otago Peninsula (Sturrock and Tompkins, 2007) using that assay have been difficult to validate.

The merozoite surface protein 1 (MSP-1) is one of the most characterized malaria proteins in human malaria and has been identified in various *Plasmodium* spp. (Polley et al., 2005; Birkenmeyer et al., 2010; Priest et al., 2018). It plays essential roles during the blood-stage development of the parasites, and is expressed abundantly on the merozoite cell surface of *Plasmodium* spp. (Hellgren et al., 2013; Lin et al., 2016). MSP-1 is the primary target of the host immune system and elicits humoral immune response during *Plasmodium* spp. infection and has thus been extensively studied as a malaria vaccine candidate (Priest et al., 2018; Blank et al., 2020). MSP-1 has been used successfully in studies for surveillance of immunity antibodies against human malaria and for understanding the epidemiology of the disease (Barry et al., 2009; Hui and Hashimoto, 2007; Ngoundou-Landji et al., 2010; Noranate et al., 2009; Tanabe et al., 2007; Pacheco et al., 2012). Therefore, in the present study, we endeavored to develop an ELISA diagnostic assay based on the merozoite surface protein 1 (MSP-1) of *P. relictum* isolate SGS1 (a potentially pathogenic species in penguins) that can facilitate the specific detection and relative quantification of antibodies in *P. relictum* SGS1-infected birds.

## 2 Materials and methods

### 2.1 Sera samples

Sera samples were obtained from 11 African black-footed penguins (*Spheniscus demersus*) at the Baltimore zoo that are typically allowed in an outdoor exhibit area from May to October. From November to February, the penguins are maintained indoors in a mosquito-free environment. The penguins sampled were identified with the following designated numbers: 8776, 8778, 8780, 8783, 8784, 8785, 8786, 8789, 8790 at the Baltimore Zoo (Maryland, USA). Each penguin was sampled every 7 days between 03/10/2020 and 11/18/2020, and the sera cryopreserved at −80 °C until use.

### 2.2 Cloning of *P. relictum* MSP-1 coding sequence

A 1476 bp long truncated coding sequence of the *P. relictum* isolate SGS1 MSP-1 (starting from the first codon) based on the gene reported in GenBank (Accession number KC969175.1) was synthesized by Integrated DNA Technologies (IDT, USA). The synthesized gene fragment was supplied by IDT cloned in pUCIDT (AMP) vector and lyophilized. Before use, the DNA was reconstituted in sterile molecular-grade nuclease-free water. The primer pair used for PCR-amplification of the truncated MSP-1 coding gene fragment for directional cloning in the pET15b expression vector (Novagen) in-frame with the N-terminal hexahistidine tag (His-tag) was 5′-*CTCGAG***ATG**ACAAATTCTTTTGGTAAAC-3′ (Forward, with the *XhoI* restriction site italicized and start codon in bold) and 5′-*GGATCC***TTA**TTCTGTATATTTGTTAATCT-3′ (Reverse, with the *BamHI* site italicized and stop codon in bold). The truncated MSP-1 gene was amplified by PCR using high fidelity DNA polymerase (Affymetrix), and T/A-cloned into the pGEMT-Easy vector (Promega). After propagation of the recombinant vector in *E. coli* and plasmid extraction, the MSP-1 coding sequence was excised by dual *XhoI/BamHI* restriction enzyme digestion and sub-cloned at *XhoI/BamHI* site of the pET15b expression vector in-frame with the His-tag at the N-terminal. The recombinant pET15b vector was sequenced to confirm identity of the gene fragment insert, and the recombinant vector transformed into protein expression *E. coli*, BL21 (DE3) pLysS (Novagen).

### 2.3 Expression and purification of recombinant MSP-1

The transformed MSP-1 protein expression *E. coli* (strain BL21 (DE3) pLysS) was cultured at 37 °C in Luria broth medium containing 100 μg/mL ampicillin and 35 μg/mL chloramphenicol until the culture reached A_600_ of about 0.6, after which protein expression was induced by the addition of 1 mM isopropyl-β-d-thiogalactopyranoside (IPTG) and cultured for a further 3 h. Bacteria were harvested by centrifugation and resuspended in lysis buffer (50 mM NaH_2_PO_4_, 300 mM NaCl, 10 mM Imidazole, pH 8.0) containing 1x EDTA-free protease inhibitor cocktail (Thermo Fisher), 600 units benzonase (EMD Millipore), 30 kU r-lysozyme (EMD Millipore), 0.5% sodium N-lauroylsarcosinate (sarkosyl), and 5% glycerol. The suspension was sonicated on ice for 5 min using 10 s discontinuous cycles. The lysate was clarified by centrifugation, and the supernatant loaded onto a column containing Ni-NTA His-bind resin (EMD Millipore) pre-equilibrated in lysis buffer. After allowing the resin to settle, the column was let to flow and the resin washed three times with a buffer containing 50 mM NaH_2_PO_4_, 300 mM NaCl, 20 mM Imidazole, pH 8.0. The recombinant MSP-1 protein was eluted from the column with elution buffer (50 mM NaH2PO4, 300 mM NaCl, 250 mM Imidazole, pH 8.0) containing 0.5% sarkosyl, and 5% glycerol. Eluted proteins were dialyzed in a buffer containing 5 mM Hepes-NaOH (pH 7.8) and 0.5 mM DTT, and concentrated using protein concentrators (Pierce). The purified protein was analyzed by SDS-PAGE to determine the purity, and protein concentration was measured using Coomassie (Bradford) protein assay kit (Thermo Scientific).

### 2.4 Recombinant MSP-1 biotinylation

Biotin was linked to MSP-1 using a Biotin Protein Labeling Kit (Roche) following the manufacturer's instructions. The purified recombinant MSP-1 protein solution was subjected to buffer exchange with PBS by centrifugation using Microcon centrifugal filters (10K MWCO, Millipore Sigma). Based on the protocol, the molar ratio between a 50 kDa protein and Biotin-7-NHS (D-Biotinoyl-ε-aminocaproic acid-N-hydroxysuccinimide ester, MW = 454.5) is 1:10. Therefore, about 1 mg of purified recombinant MSP-1 protein was added to 1 mL PBS, followed by the addition of 0.09 mg freshly prepared Biotin-7-NHS (D-Biotinoyl-ε-aminocaproic acid-N-hydroxysuccinimide ester). The mixture was incubated at room temperature for 2 h with gentle agitation to facilitate free amino groups of MSP-1 to react with D-Biotinoyl-ε-aminocaproic acid-N-hydroxysuccinimide ester (biotin-7-NHS) and form stable amide bonds. The reaction mixture was then applied to a pre-prepared Sephadex G-25 column to remove the residual non-reacted biotin-7-NHS by gel filtration. After letting the reaction mixture flow through the column, the biotin-labeled protein was eluted with 3.5 mL PBS and stored at 4 °C until use.

### 2.5 Verification of biotinylation of recombinant MSP-1 protein

To confirm the biotinylation of recombinant MSP-1 protein, 25 μL of the biotinylation eluate protein (∼300 ng/μL) was added to a 384-well polystyrene microtiter plate in triplicate and incubated at 4 °C overnight with the plate sealed with parafilm to prevent evaporation. A blank control containing 25 μL PBS instead of MSP-1-biotinylated protein, and a negative control with 25 μL unlabeled MSP-1 protein (∼300 ng/μL) were also included. Following incubation, the solutions were decanted, and the residual unbound protein removed by washing each well three times with 50 μL 1 × PBS with 1% Tween 20 (PBST). About 50 μL of 2% bovine serum albumin (BSA) in PBST was then added to each well and incubated for 1 h at 25 °C to block the non-specific binding, followed by washing three times with 100 μL PBST. Next, 25 μL of streptavidin-alkaline phosphatase (1:1000 dilution) (R&D Systems) was added to each well and incubated for 1 h at 25 °C. The unbound streptavidin-alkaline phosphatase was removed by washing three times with 25 μL PBST, followed by addition of 25 μL of p-Nitrophenyl Phosphate Disodium salt (PNPP) (Thermo Fisher). A positive control containing 25 μL streptavidin-alkaline phosphatase and 25 μL PNPP was prepared at the same time. After 2 h of incubation at 25 °C, 50 μL of 2N NaOH was added to each well to stop the reaction, and the absorbance measured at 405 nm wavelength using a SpectraMax iD5 Multi-Mode Microplate Reader (Molecular Devices).

### 2.6 Enzyme-linked immunosorbent assay

To develop an MSP-1 capture antigen-based direct enzyme-linked immunosorbent assay (ELISA) specific for the detection of anti-*P. relictum* SGS1 MSP-1 antibody in serum, 25 μL of serum from a confirmed (by microscopic analysis of Giemsa-stained thin blood smears) *Plasmodium*-infected penguin was added to a 384-well polystyrene microtiter plate in triplicate and incubated overnight at 4 °C. A negative control containing 25 μL of PBS was also included. After washing three times with 50 μL PBST, the wells were blocked with 50 μL of 2% BSA in PBST for 1 h at 25 °C to prevent nonspecific binding, followed by three washes with 100 μL PBST. Next, 25 μL of biotinylated recombinant MSP-1 protein (∼300 ng/μL) was added to each well, and the plate incubated at 25 °C for 2 h. Blank controls incubated with 25 μL of unlabeled recombinant MSP-1 protein (300 ng/μL) were included and used for background normalization. After incubation, wells were washed three times with 50 μL PBST, followed by addition of 25 μL of streptavidin-alkaline phosphatase (1000x dilution) and incubation for 1 h at 25 °C. Following three washes with 50 μL of PBST, 25 μL of PNPP (substrate for streptavidin-alkaline phosphatase) was added to each well. A positive control containing 25 μL of streptavidin-alkaline phosphatase with 25 μL PNPP was included. After 2 h of incubation at 25 °C, the reaction was stopped by adding 50 μL of 2N NaOH, and the absorbance measured at 405 nm wavelength using a SpectraMax iD5 Multi-Mode Microplate Reader (Molecular Devices).

### 2.7 Optimization of concentration of biotinylated recombinant MSP-1 protein and sera

To determine the optimal concentration of MSP-1 biotinylated protein for coating the wells for the ELISA, the biotinylated MSP-1 protein was serially diluted from 10^0^ to 10^7^ in PBS. The various dilutions were then used in the ELISA described above with the test sera maintained undiluted. In a similar manner, the *Plasmodium*-positive test penguin serum sample was serially diluted from 10^0^ to 10^7^ in PBS and used in the ELISA while maintaining the biotinylated protein undiluted at 300 ng/μL.

### 2.8 Analysis of specificity of the assay

To determine the specificity of the ELISA, the reactivity of the MSP-1 antigen to sera (Sigma) from avian (chickens) uninfected with *P. relictum* in comparison to *Plasmodium-*infected penguin's serum was analyzed. The chicken and penguin sera were serially diluted from 10^0^ to 10^7^ and used as test sera in the ELISA described above while keeping the concentration of the antigen (biotinylated MSP-1 protein) at 300 ng/μL.

### 2.9 Analysis of penguin sera test samples

Undiluted sera samples from penguins collected at various times of the year were used as test sera in the ELISA described above with the concentration of the antigen (biotinylated MSP-1 protein) maintained at 300 ng/μL.

### 2.10 Analysis of the sensitivity of the assay

Sera samples from three penguins (8776, 8784, 8783) that tested positive in the initial analysis of the test samples described above were used for the sensitivity test. The sera samples were serially diluted from 10^0^ to 10^7^ with PBS. The concentration of the biotinylated MSP-1 protein used was fixed at 300 ng/μ. The ELISA was done as described above.

### 2.11 Calculation of negative or positive cut-off point

For diagnostic ELISA tests, a cut-off value is essential for distinguishing between positive and negative samples and is commonly computed based on the mean absorbance values at 405 nm wavelength from known negative or positive controls (Minic and Zivkovic, 2020). The cut-off point in this study was determined using the Chang-point statistical analysis which detects points in a series where changes have occurred (Lardeux et al., 2016). Briefly, absorbance values of all the tested samples were arranged ascendingly and analyzed using the R package “changepoint” (Killick and Eckley, 2014). Normally, if the absorbance values from ELISA are arranged in ascending order, the negative samples (if they exist) would have lower absorbance values, while the positive samples would have higher absorbance values. The increase in the values will be irregular if positive samples exist in the series since positive samples are supposed to be “different” from the negative ones. The change-point analysis is designed to identify this difference and locate the value where this change occurs in the series. Therefore, the identified value would be the specific cut-off value that distinguishes samples with undetectable anti-*Plasmodium* antibodies from those with detectable antibodies in the series. In the present study, Pruned Exact Liner time (PELT) and CUSUM were used as the analysis and detection method in the change-point analysis, respectively.

### 2.12 Statistical analysis

Statistical analyses were performed using a two-tailed Student's *t*-test. *P* values of 0.05 or less were considered significant.

## 3 Results

### 3.1 Cloning and expression of recombinant MSP-1

**Fig. 1:**
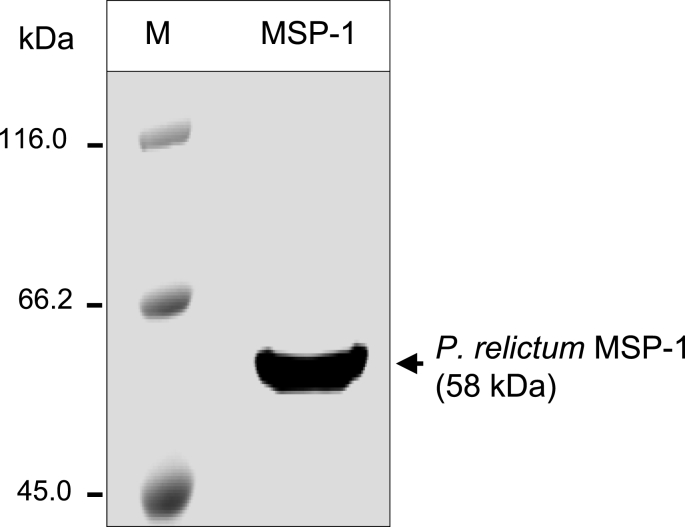
SDS-PAGE analysis of the nickel affinity column chromatography-purified truncated recombinant His-tagged *P. relictum* MSP-1 protein (∼58 kDa) stained with Coomassie blue G250. Lane M: protein ladder; lane MS-1: *P. relictum* MSP-1 recombinant protein.

SDS-PAGE analysis of the nickel-affinity chromatography-purified recombinant MSP-1 showed that it had a band size of approximately 58 kDa, consistent with its expected molecular weight, and was expressed and purified in sufficient amounts (Fig. 1). We performed blast search analysis of the truncated *P. relictum* isolate SGS1 sequence used and found that the encoded polypeptide has 99.8%, 92%, 50.4% and 35.5% identity with *P. relictum* GRW4 (GenBank accession number: KC969176.1), *P. relictum* GRW11 (GenBank accession number MZ270633), *P. gallinaceum* isolate 8A (GenBank accession number: CAH10838.1) and *P. falciparum* (GenBank accession number: BAM84577.1), respectively. This illustrates that the antigen used, while it is very similar to MSP-1 antigens of other strains within the *P. relictum* spp., it is significantly divergent from those in other *Plasmodium* spp., and hence would not likely be able to detect antibodies against other *Plasmodium* spp.

### 3.2 Biotinylation of truncated recombinant MSP-1

**Fig. 2:**
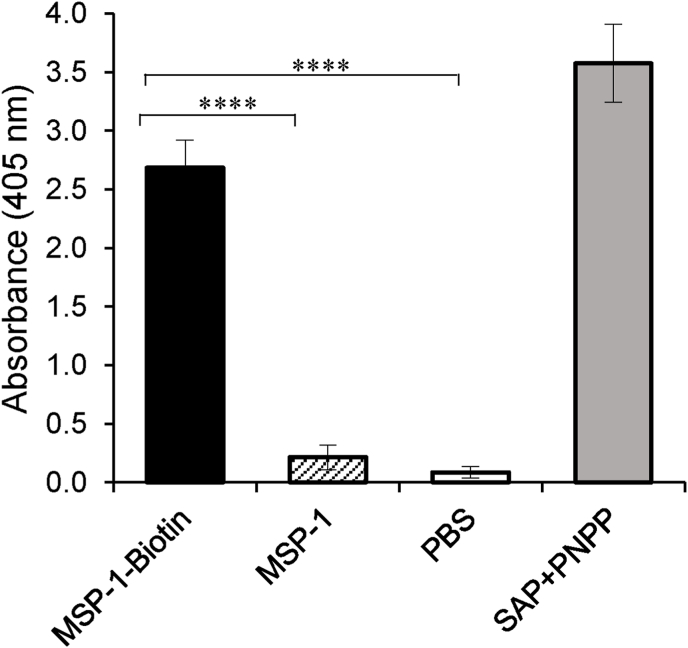
Verification of the biotinylation of recombinant *P. relictum* MSP-1. Purified recombinant *P. relictum* MSP-1 protein was labeled with biotin. Biotinylation of MSP-1 was confirmed using an enzyme (streptavidin alkaline phosphatase)-linking assay and absorbance read at 405 nm indicated the presence of biotinylated MSP-1. Black column represents biotinylated MSP-1 protein. Hatched column and white column represent negative controls containing non-biotinylated MSP-1 and PBS, respectively. Grey column represents the positive control containing streptavidin alkaline-phosphatase (SAP) and its substrate p-nitrophenyl phosphate (PNPP). Each reaction was performed in triplicate, and the data shown represent means of three independent experiments with standard error bars and levels of statistical significance (****: *P* < 0.0001).

**Fig. 3:**
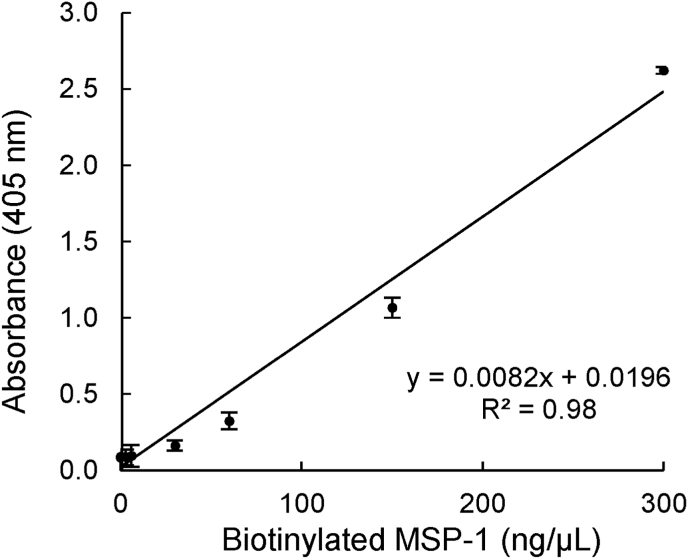
Biotin-labeled MSP-1 protein titration curve. Serially diluted (300 ng/μL to 0.003 ng/μL) biotinylated MSP-1 was used for coating the surfaces of the reaction wells overnight at 4 °C. The amount of biotinylated MSP-1 immobilized on the surface of the well was proportional to the intensity of the colored product generated which in turn was proportional to the absorbance value measured at 405 nm wavelength. Reactions were performed in triplicate, and the data shown represent means of three independent experiments with standard error bars.

Because there were no commercially available biotin-tagged anti-penguin IgG secondary antibodies for signal generation in ELISA, we endeavored to use a modified approach by tagging the truncated recombinant MSP-1 with biotin which then facilitated the biotinylation of the protein. Consistently, only the reaction wells coated with biotinylated MSP-1 showed significant (*P* < 0.0001) absorbance above background at 405 nm wavelength (A_405_ = 2.7) (Fig. 2). Titration of the biotinylated MSP-1 in the assay depicted a concentration-dependent activity of the biotin-conjugated streptavidin alkaline phosphatase with an optimum upper limit being 300 ng/μL and a minimum concentration being 8.6 ng/μL of biotinylated MSP-1 (Fig. 3).

### 3.3 Design, testing and validation of MSP-1 capture antigen-based ELISA

**Fig. 4:**
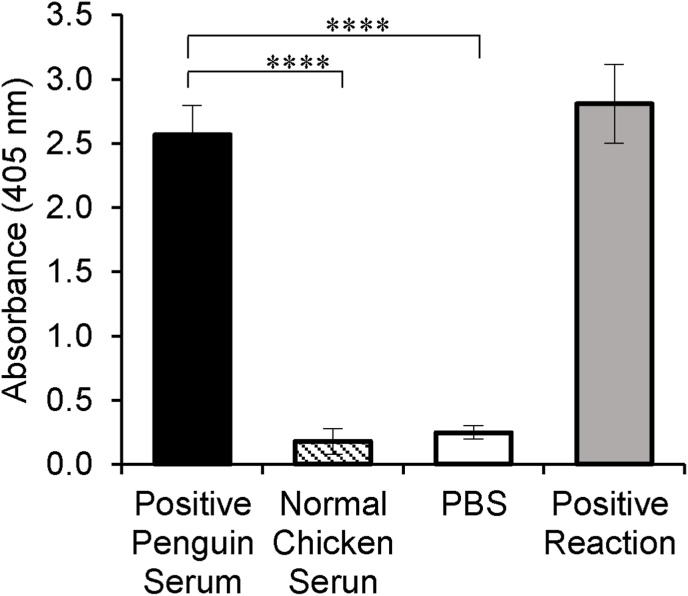
Validation of ELISA. The ELISA developed using *P. relictum* MSP-1 protein was tested with known *P. relictum* positive penguin serum collected from penguin # 8790 at week 26. Black column represents positive penguin serum. Hatched and white columns represent negative controls containing normal chicken serum and PBS, respectively. Grey column represents the positive reaction containing streptavidin alkaline-phosphatase and its substrate p-nitrophenyl phosphate. Each reaction was performed in triplicate, and the data shown represent means of three independent experiments with standard error bars and levels of statistical significance (****: *P* < 0.0001).

**Fig. 5:**
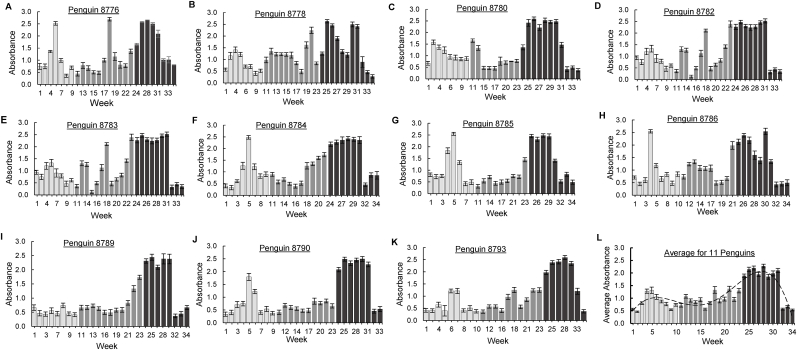
*P. relictum* MSP-1 capture antigen-based ELISA analysis of test sera from penguins. Sera samples (370 total) from eleven penguins collected from Spring through Fall season were used as test samples in the assay to determine anti-*P. relictum* antibodies level. Light grey, dark grey, and black columns represent penguin sera samples collected in Spring, Summer, and Fall, respectively. Panel A–K represents ELISA results for individual penguins' sera collected at different time points from Spring to Fall. Panel L represents average ELISA absorbances for all 11 penguins at different sampling points. Each sample was assayed in triplicate, and the data shown represent means of three independent experiments with standard error bars.

To determine the ability of the assay to detect anti-malarial antibodies in penguin sera, a sample collected from a penguin that was confirmed to be positive for *Plasmodium* infection by microscopic examination of Giemsa-stained thin blood smears was used as the test sample along with normal chicken serum and PBS as negative controls and blank, respectively. The normalized absorbances indicated that the infected penguin's serum registered significantly higher absorbances (average A_405_ = 3.00) than PBS and normal chicken serum (average A_405_ = 0.20) (Fig. 4). This indicated that the assay was sensitive enough to detect MSP-1 antibodies in the infected penguin's serum. Next, we performed the ELISA to test 370 sera samples collected from 11 different penguins over a 9 months period, from Spring through Fall. The results indicated that the levels of detectable antibodies against MSP-1 varied seasonally for individual penguins (Fig. 5). Specifically, all the penguins had detectable circulating anti-*P. relictum* MSP-1 antibodies at the beginning of Spring (before the mosquito season) (Fig. 5A–I). Overall, the antibody levels started low at the beginning of Spring, rising slightly to mid Spring, after which they declined significantly until early Summer when they started rising to reach a peak in late Fall (Fig. 5L), consistent with the seasonal prevalence patterns of the vector mosquitoes (Inumaru et al., 2021).

### 3.4 ELISA sensitivity

**Fig. 6:**
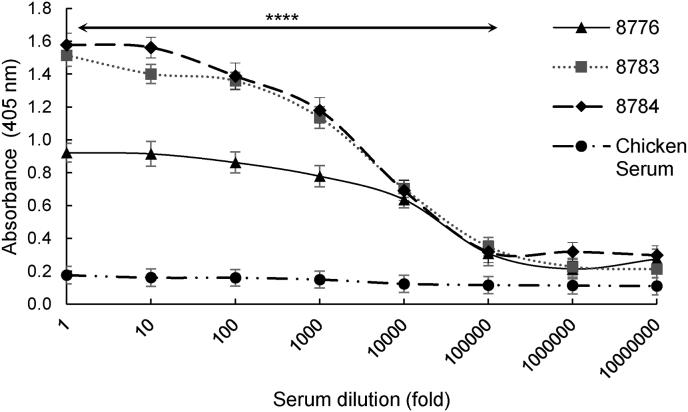
Analysis of the sensitivity and specificity of the *P. relictum* MSP-1 capture antigen-based ELISA. Serial dilutions of normal chicken serum and three sera samples from *P. relictum*-infected penguins (8776, 8783 and 8784) were used for coating the assay wells. Each dilution was performed in triplicate, and the data shown represent means of three independent experiments with standard error bars and levels of statistical significance (****: *P* < 0.0001).

To determine the sensitivity of the assay in detecting antibodies against avian malaria in infected birds, commercial normal chicken serum (as uninfected control) and three sera samples from three different penguins that were detected to have high levels of anti-MSP-1 antibodies in the initial screening were serially diluted and used as test samples in the ELISA. We found that, regardless of the dilution, the absorbances obtained from the tests using normal chicken serum were consistently around 0.200 (similar to background reading of 0.200), while those for the penguins varied in proportion to dilution, with the highest of 1.60 for undiluted sera, and the lowest of 0.20 for sera at 1 × 10^6^ fold dilution (Fig. 6).

### 3.5 Determination of ELISA cut-off point

**Fig. 7:**
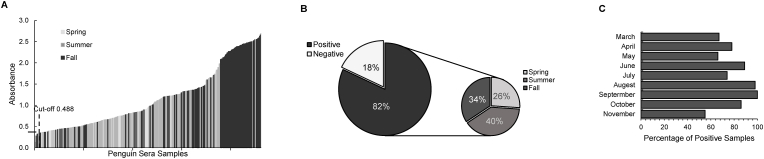
MSP-1 capture antigen-based ELISA analysis of 370 sera samples collected from 11 penguins during three consecutive seasons of Spring (March to May), Summer (June to August) and Fall (September to November). (**A**) ELISA absorbance readings for the 370 sera samples collected from Spring to Fall are arranged in ascending order. Time of collection is indicated by color. Light grey represents Spring, dark grey represents Summer, and black represents Fall. Black dashed line indicates the single cut-off point (0.488) determined by change-point analysis. (**B**) Left: percentage of positive (A_405_ ≥ 0.488) and negative (A_405_ < 0.488) sera samples; Right: distribution of positive samples in Spring (March to May), Summer (June to August), and Fall (September to November). (**C**) Percentage of penguin sera samples that tested positive (A_405_ > = 0.488) in each month from March to November.

To determine a cut-off value for the ELISA, a change-point analysis was used (Lardeux et al., 2016). Only a single change-point, located at 68 with an absorbance value of 0.4876, was detected in the series (Fig. 7A). Therefore, the “negative” and “positive” test threshold was set at 0.488, where sera samples of absorbance values higher than 0.488 were considered positive for antibodies against MSP-1. Based on the calculated cut-off, 82% of tested penguin sera samples were positive for anti-*P. relictum* SGS1 MSP-1 antibodies, while 18% were negative (Fig. 7B). Sera samples that tested positive were mainly those collected during Summer (June to August) and Fall (September to November), which account for 39% and 34% of all positive samples, respectively (Fig. 7B). Accordingly, only 27% samples were positive during Spring (March to May) (Fig. 7B).

## 4 Discussion

Effective management of penguins exposed to avian malaria is hampered by the lack of understanding and lack of characterization of the immune response of the African penguin during an active infection with avian *Plasmodium*. In the present study, we targeted the use of a merozoite surface protein 1 (MSP-1) of *P. relictum* SGS1 isolate (a potentially pathogenic species in penguins) to facilitate the specific detection and relative quantification of antibodies in *P. relictum* isolate SGS1-infected birds. MSP-1 is expressed abundantly on the merozoite cell surface of *P. relictum* (Hellgren et al., 2013; Lin et al., 2016) and has been shown to be the primary target of the host immune system, as well as elicits humoral immune response during malarial infection (Priest et al., 2018; Blank et al., 2020). Such an assay would be important for determining the *P. relictum* isolate SGS1-specific antibody prevalence in a population of penguins.

In order to develop the ELISA assay for detection of anti-*P. relictum* isolate SGS1 antibodies in penguins, because there are no commercially available tagged anti-penguin IgG secondary antibodies that can be used for signal generation, we endeavored to use a modified approach by tagging a truncated recombinant MSP-1 protein with biotin that was then used as antigen in the assay. The full open reading frame of *P. relictum* isolate SGS1 MSP-1 codes for a protein that is about 183 kDa in size. Proteins this large are difficult to express in sufficient amounts and are prone to coagulation, which is not amenable to biotinylation. Therefore, we truncated the protein to 58 kDa and found this to be abundantly expressed in *E. coli* and we could maintain it in soluble state after purification. We reasoned that a 58 kDa protein is of sufficient size to provide for the linking of several molecules of biotin to the protein, and thus can provide high assay sensitivity. Biotin is a very small molecule, which does not significantly alter the structure of the biotinylated proteins. Because biotin binds to streptavidin with extremely high affinity and specificity, we utilized the conjugation of streptavidin alkaline phosphatase to biotin, and the subsequent generation of a colored product by the enzymatic activity of the streptavidin phosphatase on the substrate PNPP in the assay. Thus, the principle of the assay was based on the interaction between a biotinylated truncated recombinant MSP-1 protein and antibodies generated against the *P. relictum* isolate SGS1 MSP-1 in the infected penguins’ sera.

The validity of the use of the biotinylated MSP-1 in the assay was tested by using serially diluted concentrations of the protein, and using test serum from a penguin that was confirmed to be infected with *Plasmodium* by microscopic examination. The findings from this analysis indicated that the assay could be used to quantify the relative levels of antibodies in test sera. We used the assay to analyze 370 field sera samples obtained from eleven penguins at the Baltimore Zoo over three seasons within a year and found that the levels of detectable antibodies against MSP-1 varied seasonally for individual penguins in concordance with the expected seasonal variations in avian malaria prevalence (Inumaru et al., 2021). The Baltimore Zoo is within the active range of the mosquito vector for avian *Plasmodium* (Beier and Stoskopf, 1980). The seasonality of the disease in penguins mainly results from the changes in population and activity of the vector mosquito. Mosquitos are most active during mid-Summer and early Fall in the Northern Hemisphere, indicating increased or high *Plasmodium* exposure that activates the immune responses of penguins (Inumaru et al., 2021).

Indeed, our ELISA results showed that 73% of the positive sera with relatively high anti-MSP-1 antibodies were collected during Summer and Fall, thus further validating the assay. These observed changes of antimalarial antibodies circulating in penguins follow the same pattern as the course of adaptive immune responses after being exposed to a specific pathogen (Ademolue and Awandare, 2018; Rogers et al., 2021). Among the sampled penguins, naive birds were most likely first exposed to mosquitoes carrying *Plasmodium* parasites at the beginning of Spring, which activated primary adaptive immune responses. With increasing mosquito activity in early to mid-Summer, subsequent parasite challenges triggered secondary adaptive immune responses, which led to higher antibody titers as illustrated in the ELISA results. Corroboratively, when we analyzed the sensitivity of the assay by titrating the penguin sera that had tested positive, we found that the signal intensity generated was serum concentration-dependent, thus validating the ability of the assay to detect and relatively quantify levels of antimalarial antibodies in penguin sera. By comparing antibody levels between penguins, the ones with less active immune responses would be identified, which would help plan prophylaxis or treatment. Only individuals with relatively low antibody levels may need treatment to help them survive the malaria season, as unnecessary treatment may interfere with the development of protective immunity, which would leave the penguins more susceptible in the following season (Grilo et al., 2016).

## Ethics

All experiments involving the use of animals in this study were carried out in strict compliance with the recommendations and guidelines of the United States Department of Agriculture Animal Welfare Act and the National Institute of Health Public Health Service Policy on the Humane Care and Use of Animals. The protocol number 20093 was approved by the University of Illinois Institutional Animal Care and Use Committee for conducting research with penguins. All efforts were made to reduce the pain and suffering of the birds.

## Declaration of competing interest

None.
